# ELABELA: A Potential Therapeutic Target for Ischemia–Reperfusion Injury

**DOI:** 10.3390/biom16020301

**Published:** 2026-02-14

**Authors:** Wan Li, Cuicui Meng, Shujun Yang, Yibei Xie, Zijie Huang, Tong Wang

**Affiliations:** Department of Emergency, The Eighth Affiliated Hospital of Sun Yat-sen University, Shenzhen 518033, China; liwan8@mail2.sysu.edu.cn (W.L.); mengcc@mail2.sysu.edu.cn (C.M.); yangshj26@mail2.sysu.edu.cn (S.Y.); xieyb25@mail2.sysu.edu.cn (Y.X.); huangzj56@mail2.sysu.edu.cn (Z.H.)

**Keywords:** ELABELA, ischemia–reperfusion injury, oxidative stress, inflammatory response, apoptosis, fibrosis, angiogenesis

## Abstract

Ischemia–reperfusion injury (IRI) refers to a paradoxical pathological process in which ischemic tissues or organs sustain further damage upon the restoration of blood flow and is clinically commonly observed in the peripheral vascular systems and various vital organs. In 2013, the endogenous peptidic hormone ELABELA (ELA) was identified as a novel native ligand of the APJ G protein-coupled receptor, alongside apelin. ELA is constitutively expressed in a range of adult and embryonic tissues and plays a role in numerous physiological and pathological processes. In recent years, a rapidly growing body of research has highlighted the cytoprotective properties of ELA against IRI. In this review, we synthesize current research on the protective role of ELA in mitigating IRI across multiple organ systems.

## 1. Introduction

In the conventional paradigm of understanding, restoring blood flow to ischemic tissue is expected to promote healing; however, clinical reality often contradicts this assumption. Rather than ameliorating the damage, reperfusion can exacerbate injury in previously ischemic tissues—a phenomenon known as ischemia–reperfusion injury (IRI) [1]. The pathophysiology of IRI is complex, involving a cascade of interrelated mechanisms. During the ischemic phase, cells succumb to energy depletion, resulting in widespread dysfunction and structural damage. Although reperfusion is essential for rescuing these ischemic tissues, it simultaneously unleashes a barrage of deleterious events—both locally and in remote organs—that can amplify the initial injury [2,3].

**Figure 1:**
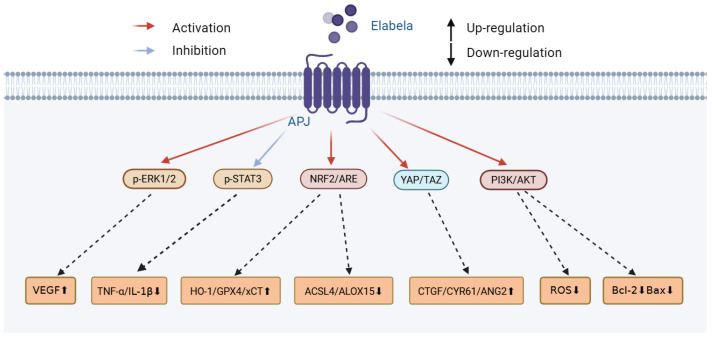
ELABELA activates APJ and its related pathways.

Historically, the APJ receptor was assumed to be activated solely by apelin; however, between 2013 and 2014, two independent teams identified a second endogenous ligand: ELABELA (ELA, also known as toddler) (Figure 1) [4,5]. The APELA gene encodes this peptidic hormone, which is proteolytically processed into multiple bioactive forms [4,5]. Apelin and ELA both activate the G protein-coupled APJ receptor but induce distinct signaling patterns [6]. ELA is constitutively expressed in both adult and embryonic tissues, with its levels being highest in vascular endothelium, heart, kidneys, placenta, and circulating plasma [7,8,9]. Moreover, ELA exhibits broad cytoprotective activities, including having anti-inflammatory, antioxidant, and anti-fibrotic properties, and modulates apoptosis, ferroptosis, and angiogenesis [10,11,12].

Over the past decade, interest in ELA’s capacity to mitigate IRI has surged; an expanding body of pre-clinical work now documents its organ-protective effects in the brain, heart, kidney, liver, and other tissues [13,14,15,16,17,18,19,20,21]. In this review, we synthesize the literature to appraise ELA’s therapeutic potential against IRI across multiple organ systems.

## 2. Ischemia–Reperfusion Injury

### 2.1. Clinical Context of Ischemia–Reperfusion Injury

Restoring blood flow to an ischemic organ is non-negotiable, yet the very act of reperfusion often unleashes a second, covert assault. Primary percutaneous coronary intervention (PCI) is performed in almost half of the patients with acute ST-segment elevation myocardial infarction (STEMI) [22], and within hours, IRI can enlarge the final infarct by up to 50% [23]. Liver transplant offers an even starker lesson: graft IRI is documented in 87.4% of recipients, and the resulting oxidative stress and systemic inflammatory wave can propagate to the lungs, kidneys, and heart, culminating in multiple organ dysfunction syndrome [24,25]. Analogously, after effective mechanical thrombectomy for acute stroke, almost 46.9 percent of patients succumb—not to the original occlusion, but to reperfusion-driven expansion of the cerebral lesion [26]. Across disciplines, life-saving revascularization casts the shared pathological shadow of IRI over every organ it rescues, and so its prevention has become a clinical imperative.

### 2.2. Dual-Phase Pathological Mechanism of Ischemia–Reperfusion Injury

The pathophysiology of IRI involves two phases, namely, the ischemic phase and the subsequent reperfusion phase, the latter of which has a hallmark biphasic characteristic [2,3]. Fully understanding the biphase process is critical for identifying tractable therapeutic targets.

#### 2.2.1. Pathological Mechanisms in the Ischemic Phase

The instant that blood flow ceases, oxygen tension collapses and oxidative phosphorylation stalls. Cells are forced to shift from efficient aerobic respiration to anaerobic glycolysis, a form of metabolic rescue that cannot be sustained. ATP generation plummets, while lactate and protons accumulate, driving the intracellular pH below 6.8 and triggering early acidosis [27,28]. ATP depletion and acidosis converge to cripple Na^+^/K^+^-ATPase and Ca^2+^ pumps, halting the export of Na^+^ and Ca^2+^. Rising intracellular Na^+^ osmotically draws water, producing edema, while acidosis further activates the Na^+^ exchanger to amplify its loading. The accumulation of Na^+^ reverses the Na^+^/Ca^2+^ exchanger, flooding the cytosol with Ca^2+^, and endoplasmic reticulum uptake and release mechanisms stall. The resulting cytosolic and mitochondrial Ca^2+^ overload activates calpains and other Ca^2+^-dependent proteases of lysosomal membranes, releasing cathepsins and other hydrolases into the cytoplasm, which cleave cytoskeletal and contractile proteins, dismantling the cellular architecture and precipitating death by necrosis and apoptosis [29,30,31,32,33].

#### 2.2.2. Pathological Mechanisms in the Reperfusion Phase

In the reperfusion stage, the sudden re-oxygenation of ischemic tissues causes a volcanic burst of reactive oxygen species (ROS), triggering oxidative stress [29]. With regard to provenance, early ROS are generated from the mitochondrion within the first hours after reperfusion, whereas the later wave—peaking at about 24 h—comes from infiltrating leukocytes and macrophages [34]. ROS attack DNA, protein oxidation, and lipids, and enhance structural disruption and cellular damage through pathways that include triggering inflammatory cascades, perforating mitochondria, and disrupting intracellular calcium homeostasis [35,36,37]. When this ROS surge coincides with the Ca^2+^ overload inherited from ischemia, the two insults trigger the process of opening mitochondrial permeability transition pores (mPTPs)—a process held in check during ischemia only by the prevailing acidic milieu [38,39]. Once the mPTPs open, the mitochondrial membrane potential collapses, ATP synthesis grinds to a halt, and the cell is energetically bankrupted, committing it to apoptosis or necrosis [40,41]. While the organelle fails, injured or necrotic cells emit damage-associated molecular patterns (DAMPs) that alert the innate immune system. These molecular beacons trigger an immediate sterile inflammatory reaction and recruit neutrophils and monocytes to the injury site [42,43]. These newly recruited proinflammatory cytokines include tumor necrosis factor-1 (TNF-1), interleukin-1 (IL-1), interleukin-6 (IL-6), as well as a pantheon of downstream mediators—and simultaneously ignite pyroptosis, necroptosis, and additional ROS generation, triggering tissue damage [29,44]. Pattern recognition receptors like Toll-like receptors sense DAMPs and propagate innate immune responses, indirectly regulating adaptive immunity, and all three arms of the complement system (classical, alternative, and lectin) are brought online. Together, these inflammatory signals form a self-amplifying loop that continually intensifies inflammatory- and tissue-related injury [42,45,46]. Reperfusion modalities of cell deaths are also complex; apoptosis, necroptosis, pyroptosis, and ferroptosis can ignite in parallel, their signaling networks crisscrossing to magnify tissue destruction and injury [47,48,49]. At the microvascular level, the same inflammatory milieu dismantles the endothelial glycocalyx; platelets and leukocytes clasp to one another and trapped neutrophils plug capillaries, triggering the “no-reflow” effect—producing an open macro-vessel with stagnant microcirculation [50,51]. This secondary vascular failure not only weakens the effectiveness of reperfusion therapy but also predicts larger infarcts and worse clinical outcomes [52,53]. Fibrosis is a late and persistent tissue remodeling response after ischemic reperfusion injury, characterized by the replacement of damaged tissue by excessive fibrosive tissue. Initially, it is an attempt to restore tissue integrity through a reparative mechanism, but it often converts to pathological changes due to signaling over-activation, leading to adverse outcomes closely associated with patient clinical prognosis [1].

## 3. Mechanisms Underlying the Protective Effects of ELA

### 3.1. Introduction to ELA

**Figure 2:**
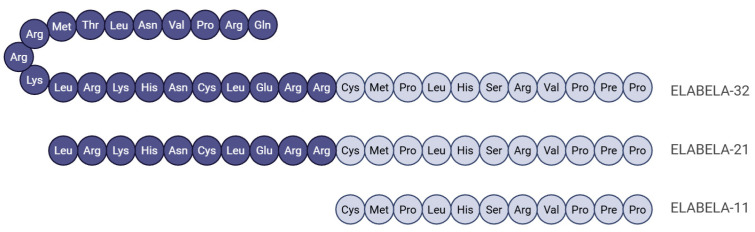
After processing ELA, ELA-32, ELA-21, and ELA-11 are produced.

ELA is a newly described endogenous peptidic hormone that partners with apelin as a core component of the apelinergic system, jointly orchestrating key physiological functions [54]. APJ was discovered in 1993 and belongs to the G protein-coupled receptor (GPCR) family. As its endogenous ligand remained unknown for a long period, this receptor was once classified as an orphan receptor. In 1998, Apelin was identified as the first endogenous ligand for APJ and was subsequently regarded as the sole ligand for this receptor for over a decade, until the discovery of ELA [55]. ELA was independently discovered in 2013 and 2014, the peptide received two names: Chng et al. (2013) christened the gene ELA—an acronym for “Endoderm late arrival”—after observing delayed gastrulation in zebrafish knockouts, whereas Pauli et al. (2014) called it Toddler to emphasize impaired embryonic cell migration when the gene is absent [4,5]. Once dismissed as a non-coding region in the genomes of fishes and humans, the locus was later shown to encode a 54-amino-acid precursor peptide that is cleaved into the mature fragments ELA-32, ELA-21, and ELA-11 (Figure 2). Each isoform binds and activates the APJ receptor, and their actions are abolished by APJ antagonists [56,57]. In human plasma and renal homogenates, ELA-32 is further trimmed to shorter, still-active species such as ELA-16, ELA-19, and ELA-20, generating a combinatorial peptide code that fine-tunes multiple physiological outputs [58]. Although both ELA and apelin can activate a G protein-coupled APJ receptor that is involved in a variety of physiologies, they show significant differences in binding mode and signaling preference: Elabela-32 is a strong β-arrestin-biased ligand, while Elabela-21 favors the G protein pathway; Apelin-17 favors β-arrestin, while Apelin-36 is more balanced. These differences provide an important basis for developing highly selective J-targeted drugs [6].

ELA expression has been documented in both adult and embryonic tissues, with the highest levels in the vascular endothelium, heart, kidneys, and placenta; circulating forms are readily detectable in plasma [7,8,9]. ELA plays important physiological roles both in the embryo phase and in adult life. Zebrafish lacking ELA exhibit profound cardiac malformations, including cardiac agenesis—a phenotype that mirrors APJ ablation and deletion—confirming ELA as a bona fide APJ ligand [4]. Similarly, murine ELA deficiency produces embryonic cardiovascular anomalies, placental insufficiency, and overt preeclampsia-like signs in pregnant dams [9,59]. Beyond development, ELA maintains the self-renewal and pluripotency of human embryonic stem cells, inhibits apoptosis, and promotes initial cell migration and organogenesis [60]. In adults, tonic ELA-APJ signaling continues to balance cardiovascular and renal homeostasis, endowing the heart, kidneys, and other organs with cytoprotective reserves against pathological stress [61,62,63].

### 3.2. Protective Mechanisms of ELA

#### 3.2.1. Mitigation of Oxidative Stress

Low-level oxidants are obligatory signaling currencies, yet their presence in excess tips the scale toward injury. Oxidative stress arises when pro-oxidants outnumber antioxidants, distorting redox circuits and causing molecular impairment [64,65]. Several studies in recent years have shown that ELA acts as a potent antioxidant across various disease models, and curbs glucose-containing ROS accumulation and resulting DNA oxidative lesions by suppressing the TRAF1-dependent NF-κB axis [66]. In DOCA-salt hypertensive kidneys, it silences the NADPH-oxidase/ROS/NLRP3 inflammasome pathway, averting oxidative injury [67]. It also triggers Krüppel-like Factor 15 (KLF15), restoring intracellular glutathione (GSH) and boosting the antioxidant enzyme Glutathione peroxidase 4 (GPX4), thereby neutralizing doxorubicin-generated ROS and curbing oxidative stress [68]. In hypertensive hearts, ELA quells IL-6/STAT3 signals while upregulating the xCT/GPX4 and eNOS/Nrf2 axes, simultaneously inhibiting ROS production and reinforcing cellular antioxidant defenses [11].

#### 3.2.2. Suppression of Inflammatory Response

Inflammation is the body’s innate defense program, involving the dispatch of immune cells and mediators to eliminate pathogens, promote repair, and adapt to stress [69]. However, every system has a tipping point; once inflammation is over-activated, tissue damage and organ dysfunction ensue [70]. When inflammation becomes out of control, ELA steps in to prevent it, with one key mechanism to achieve this, involving activation of the fibroblast growth factor 21 (FGF21), which upregulates angiotensin-converting enzyme 2 (ACE2); elevated ACE2, in turn, blunts angiotensin II-driven transcription of proinflammatory cytokines, extinguishing the inflammatory flare [71]. ELA suppresses TRAF1 through its cognate receptor APJ, inhibiting the phosphorylation and subsequent activation of the NF-1 pathway to eventually suppress proinflammatory cytokine synthesis and curb inflammation [66]. In sepsis models, ELA averts macrophage pyroptosis by preventing caspase-1- and gasdermin-D-mediated pore formation, thereby restraining IL-1β release while preserving bacterial clearance [72].

#### 3.2.3. Regulation of Apoptosis

Apoptosis is the cell’s scheduled exit strategy, representing an ATP-dependent, tightly orchestrated dismantling that maintains tissue identity while purging damaged or dangerous cells. Yet this is a double-edged sword: when thresholds are miscalibrated or checkpoints overridden, excessive or insufficient apoptosis becomes a driving force in myriad diseases [73,74]. Emerging research has reinforced the ability of ELA to coordinate critical signal transduction and apoptosis-related molecules, thereby regulating apoptosis in a wide range of cell types. In murine diabetic nephropathy, ELA triggers the PI3K/Akt/mTOR pathway to shift B-cell lymphoma 2 (Bcl-2) family balance toward survival and suppress caspase-3 appearance, ultimately reducing podocyte apoptosis [75]. Likewise, in bone marrow mesenchymal stem cells, ELA induces phosphorylation of both AKT and ERK1/2, upregulates Bcl-2, and creates a comparable antiapoptotic shield [76]. It triggers the FGF21-ACE2 signaling pathway in rat adventitial fibroblasts, which significantly inhibits angiotensin II-progeny-induced apoptosis [71]. In neurons, ligand-occupied APJ upregulates miR-124-3p, which silences C-terminal domain small phosphatase 1 (CTDSP1) and alleviates its brake on AKT phosphorylation; the ensuing AKT signaling pathway, in turn, delivers a potent antiapoptotic signal that protects neurons from ischemic death [77].

#### 3.2.4. Inhibition of Ferroptosis

Ferroptosis—an iron-dependent, nonapoptotic form of regulated cell death—occurs when lipid peroxides accumulate beyond the antioxidant system’s capacity to neutralize them, ultimately rupturing the plasma membrane and causing cell death [78,79]. Recent work has shown that ELA thwarts ferroptosis on three converging fronts: First, it transcriptionally upregulates the xCT/glutathione peroxidase 4 (GPX4) pathway and thereby curbs lipid peroxide buildup and free-iron accumulation [11,80]. Second, ELA blocks ferritinophagy, increases the levels of ferritin heavy chain (FTH1), and shrinks the labile iron pool; this inhibits ferroptosis death in placental trophoblasts and ultimately alleviates the pathological characteristics of preeclampsia [81]. Third, ELA triggers KLF15, which boosts Nrf2/SLC7A11/GPX4, further enhancing the production of glutathione and preventing the process of lipid-peroxidation-driven rupture [68].

#### 3.2.5. Inhibition of Fibrosis

Fibrosis refers to the mechanism through which excess depositions of extracellular matrix elements result in the disorganization of normal tissue structure and the eventual dysfunction of the organ as a consequence of continued injury or chronic inflammation stimuli [82,83]. The pathogenic processes of fibrosis are not confined to the triggers of reorganization, tissue sclerosis, and functional failure of organs, but may also further develop to end organ disease, which is a leading cause of death in many chronic diseases; it has been estimated that fibrosis causes 45% of all deaths in developed countries [82,83]. Accumulating evidence positions ELA as a broad-spectrum anti-fibrotic agent; it suppresses IL-6/STAT3 signaling and blocks ferroptosis in cardiac microvascular endothelial cells (CMVECs), thereby restraining the activation, proliferation, and migration of cardiac fibroblasts and ultimately alleviating cardiac fibrosis in hypertensive mice [11]. ELA-32 suppresses TGF-2/SMAD2/3, ERK1/2, and AKT signaling in TGF-β1-stimulated human peritoneal mesothelial cells, preventing epithelial-to-mesenchymal transition, indicating its inactive anti-fibrotic ability in the peritoneum [84].

#### 3.2.6. Promotion of Angiogenesis

Angiogenesis (sprouting of new blood vessels from existing ones) plays a crucial role in embryonic development and growth and tissue healing, yet its dysregulation fuels aberrant, often pathological vascular growth that underlies disorders ranging from tumor progression to ischemic tissue dysfunction [85]. A vast amount of research supports the nexus between ELA and angiogenesis. ELA triggers ERK1/2 signal transduction, stabilizes hypoxia-inducible factor-1a (HIF-1a), and enhances the release of VEGF, thereby driving classic angiogenic sprouting to occur [17]. Separately and independently of VEGF, ELA-APJ act as a midline chemoattractant that guides angioblast migration during early embryogenesis, ensuring proper vascular assembly before primary vessels form [86]. ELA amplifies the VEGFR2/AKT signaling pathway, accelerating endothelial cell proliferation and tube formation and alleviating the angiogenesis process in post-ischemic conditions [87,88]. The ligation of ELA via the APJ receptor additionally recruits the VEGF/VEGFR2 and Jagged1/Notch3 pathways, forging a multi-pathway angiogenic program that promotes robust, orderly vascular growth [88,89].

## 4. Protective Effects of ELA in Ischemia–Reperfusion Injury of Various Organs

### 4.1. Protective Effects of ELA Against Cerebral Ischemia–Reperfusion Injury

**Figure 3:**
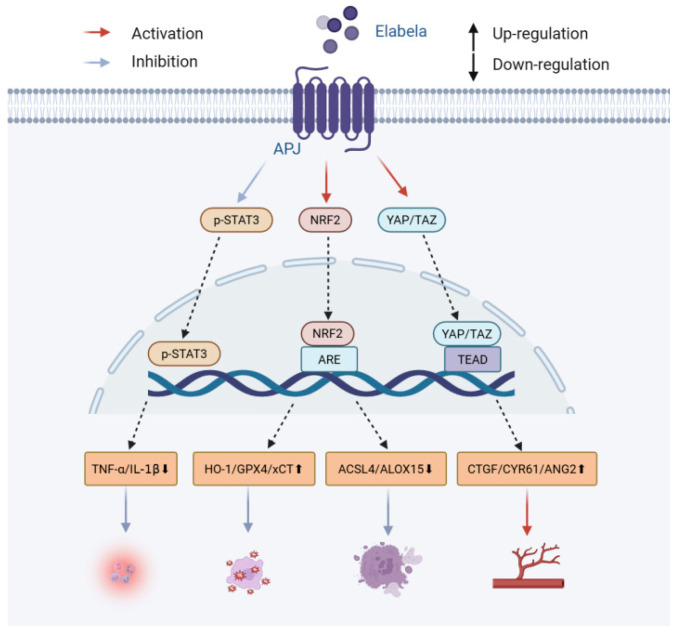
Mechanism of ELA’s protective effects against cerebral ischemia–reperfusion injury.

Cerebral IRI refers to a pathological process through which cerebral perfusion is reinstated after cerebral ischemia, amplifying tissue injury via an array of pathological processes [90]. Authors of modern studies have determined that ELA, via its cognate receptor APJ, causes pleiotropic cytoprotective impacts throughout cerebral ischemia–reperfusion damage, and displays orchestrating neuroprotective power (Figure 3). In the presence of oxidative stress, the ELA-APJ axis enhances the NRF2/ARE antioxidative signaling pathway, increasing the expression of antioxidative genes such as heme oxygenase-1; reducing lipid peroxidation byproducts, including malondialdehyde (MDA) and 4-hydroxynonenal (4-HNE); and strengthening the glutathione system, thereby neutralizing oxidative stress in the post-ischemic brain [13]. ELA-32 administration after cerebral ischemia strongly inhibits neuronal ferroptosis by reprogramming ferroptosis-related genes expression, reducing intracellular iron, and repairing mitochondrial morphology—effects that require both the APJ and NRF2 pathways [13]. In terms of both inflammation and apoptosis prevention, remote ischemic preconditioning increases the expression of ELA and APJ, reduces the abundance of proinflammatory cytokines, and suppresses the expression of caspase-3, eventually preventing neuroinflammation and neuronal apoptosis [14]. Separately, ELA binding to APJ activates the YAP/TAZ signal to promote angiogenesis, which increases the concentration of pro-angiogenic factors and enhances endothelial cell proliferation, migration, and tube formation; this promotes cerebral vascular intima neovascularization after ischemia and blood circulation recovery [15]. ELA exerts multi-stage protective effects against cerebral ischemia–reperfusion injury: during the acute phase, it directly protects neurons through anti-inflammatory, antioxidant, and anti-ferroptosis mechanisms; over the longer recovery period, it further provides sustained support for structural repair and functional recovery of brain tissue by promoting angiogenesis and collateral circulation reconstruction.

### 4.2. Protective Effects of ELA Against Cardiac Ischemia–Reperfusion Injury

**Figure 4:**
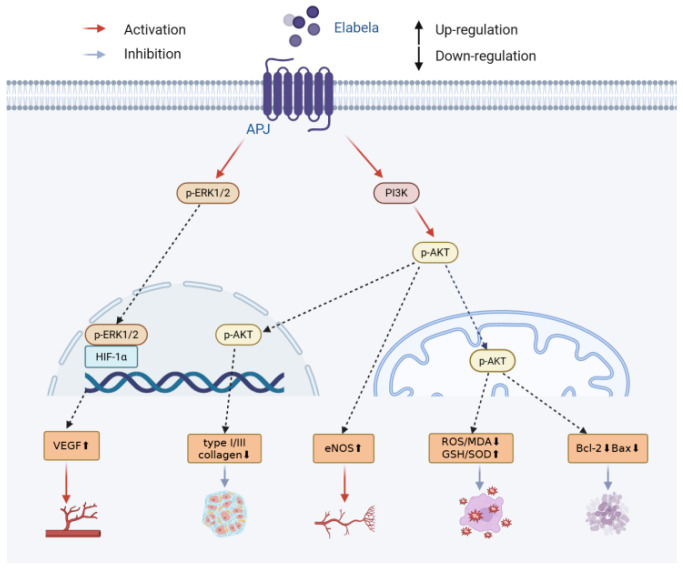
Mechanism of ELA’s protective effects against myocardial ischemia–reperfusion injury.

Myocardial IRI describes a situation in which the restoration of blood flow after ischemia does not result in complete functional and structural restoration, but leads to the expression of many pathological processes which further damage cardiomyocytes, ultimately increasing the size of infarctions and permanently limiting function recovery [91]. ELA mitigates myocardial IRI in a variety of molecular ways (Figure 4): ELA has the capability to partially activate the PI3K/AKT signal transducer, counteracting oxidative stress, which lowers the levels of ROS and MDA and increases the levels of GSH and SOD [16]. Regarding the inhibition of apoptosis, ELA suppresses this process through the mitochondrial pathway by lowering the levels of proapoptotic protein, Bax; increasing the levels of antiapoptotic protein, Bcl-2; and inhibiting cytochrome c release [16]. ELA also significantly depresses expression type I and III collagens, helping to weaken myocardial interstitial fibrosis, which also requires the activation of PI3K/AKT signals [16]. ELA also switches on the ERK1/2 signaling pathway, leading to the upregulation of HIF-1alpha and VEGF, and increases capillary density within myocardial areas, thereby enhancing blood flow to ischemic areas [17]. ELA exerts a multi-phase synergistic protective effect against myocardial ischemia–reperfusion injury by mitigating damage through its antioxidant and anti-apoptotic actions in the acute phase, and by promoting angiogenesis and exerting anti-fibrotic effects in the late phase.

### 4.3. Protective Effects of ELA Against Renal Ischemia–Reperfusion Injury

**Figure 5:**
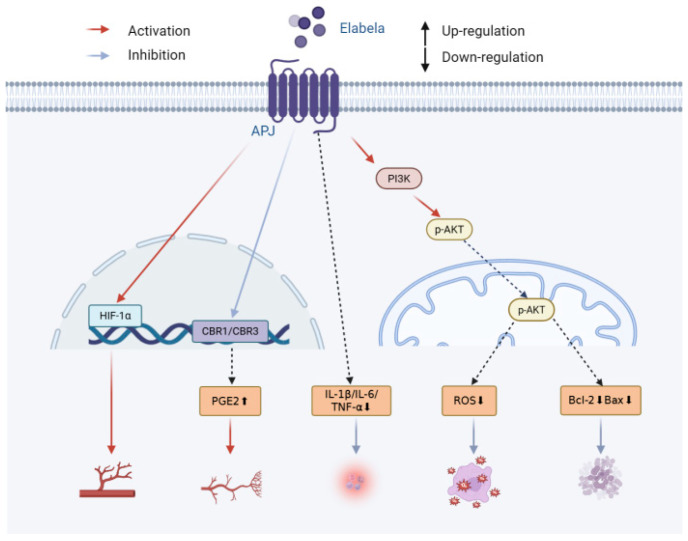
Mechanism of ELA’s protective effect on renal ischemia–reperfusion injury.

The term renal IRI is used to characterize a pathological cascade that follows temporary interruption of blood flow to the kidney, resulting in ischemia, which is followed by the restoration of blood flow and excessive production of ROS, creating strong oxidative stress and inflammatory signals and ultimately leading to cellular impairment and death. This eventually results in acute kidney injury (AKI) and renal insufficiency [92]. Previous research has outlined that, through a multitarget effect, ELA exerts pivotal renoprotective action (Figure 5). Fc-ELA-21 plays a powerful role in reducing oxidative stress and eliminating the production of ROS in the renal parenchyma, thus reducing oxidative insult [18]. Regarding inflammatory cascade inhibition, ELA prevents the expression of proinflammatory factors and limits macrophage invasion [19], and has also been shown to induce augmented neutrophil and lymphocyte infiltration into renal tubules in ELA knockout mice, clear evidence that ELA inhibits inflammatory cell trafficking and renal inflammation [20]. ELA activates the PI3K/Akt signaling axis to tilt the antiapoptotic balance toward survival—upregulating Bcl-2, decreases the proapoptotic protein Bax, and prevents the activation of caspase-3—thereby shielding epithelial cells from death [18]. In the attenuation of fibrosis, ELA suppresses fibrotic biomarkers and collagen deposition, slowing fever-induced acute kidney injury to chronic kidney disease (CKD) [19,20]. ELA-APJ signaling improves renal microcirculation by upregulating the angiogenic factors Angiopoietin 1 (Angpt1), vascular endothelial growth factor A (VEGFA), and Tie1, and increasing the microvascular density of CD31+-measurable renal microcirculation [20]. In the early stage of renal ischemia–reperfusion injury, ELA alleviates acute kidney damage through its anti-inflammatory, antioxidant, and anti-apoptotic effects. In the late stage, it delays the progression to chronic kidney disease by protecting the renal microcirculation and inhibiting fibrosis.

### 4.4. Protective Effects of ELA on Hepatic IRI and Distant Organ Involvement

Hepatic ischemia–reperfusion injury refers to an etiological process where restoration of microcirculatory blood flow following hepatic ischemia, triggered by innate and adaptive immune cascades, metabolic perturbation, oxidative stress, and activation of multiple cellular death signaling pathways, leads to further hepatocyte defects and sinuously oscillating endothelial cells, thus reducing hepatic clearance recovery [93]. ELA decreases oxidative stress biomarkers and returns levels of antioxidative molecules in the liver and its remote organs to control levels, which is caused by HI/R damage in hepatic HI/R models, and this supports its ability to decrease oxidative stress in the process [21]. In inhibiting inflammatory reactions, ELA reduces liver hepatic myeloperoxidase (MPO) activity but has little impact on pulmonary and renal MPO activity, suggesting that it inhibits local hepatic cell infiltration of inflammatory cells [21]. In the case of cellular apoptosis, short-term ELA treatment has no significant effects on caspase-3; in long-term reperfusion, it increases the expression of caspase-3 and 8-hydroxydeoxyguanosine (8-OHdG), suggesting that it can cause the death of damaged hepatocytes through its ability to promote observed apoptosis, thus leading to tissue restoration [21]. In liver fibrosis inhibition, ELA significantly reduces the activation of α-smooth muscle actin (α-SMA)-positive hepatocellular cells induced by long-term HI/R, demonstrating its anti-fibrotic effect [21]. ELA plays a protective role in the acute phase by anti-inflammatory and antioxidant effects, and in the late stage by promoting liver regeneration and repair and inhibit fibrosis to play a role in organ protection.

## 5. Limitations

Although, in this review, we summarized current literature on ELA and ischemic reperfusion injury, relevant studies are still in the preliminary stage, and the literature cited is limited. It is worth noting in particular that the study of ELA’s impact on ischemic reperfusion injury is gradually emerging, and its mechanism and potential need to be further explored. Whether ELA has any protective effect in other IRI locations like the GIT or lungs is still untested, and the precise molecular underpinnings of its hepatic protection are still fragmentary. Therefore, there is an urgent need for more high-quality studies to enrich the evidence base and illuminate the specific action pathways and clinical value. It is believed that the number of studies on ELA and IRI will continue increasing in the coming future, with ELA being a potentially productive therapeutic agent against IRI across multiple organ systems.

## 6. Conclusions

IRI is a virtually inevitable chief sequela seen in clinical practice; however, this is an ultimate clinical scenario which lacks any approved therapeutic modality. The past few years have witnessed a surge in studies revealing that the endogenous peptide ELA exerts broad, organ-spanning protection against IRI in the heart, brain, kidneys, and liver. Operating chiefly through its receptor APJ, ELA comprises a variety of modalities such as antioxidative defense, inflammatory quelling, apoptotic regulation, ferroptosis and fibrosis suppression, and angiogenesis regulation—occurring via PI3K/AKT, ERK, and Nrf2 signaling (Table 1). These concerted actions preserve microcirculatory integrity and prevent remote organ damage, positioning ELA as a promising pleiotropic guardian against ischemia–reperfusion injury.

## Figures and Tables

**Table 1:** ELA treatment in IRI. ↓: downregulation, ↑: upregulation.

Effects	Type of Disease or Condition	Mechanisms	Reference
Alleviate oxidative stress	Cerebral IRI	NRF2/ARE pathway ↑	[13]
	Myocardial IRI	PI3K/AKT pathway ↑	[16]
	Renal IRI	PI3K/AKT pathway ↑	[18]
	Hepatic IRI	ROS ↓	[21]
Alleviate the inflammatory response	Cerebral IRI	p-STAT3 pathway ↓	[14]
	Renal IRI	IL-6/TNF-α/MCP-1/ICAM-1 ↓	[19]
	Hepatic IRI	MPO ↓	[21]
Inhibit apoptosis	Cerebral IRI	Caspase-3 ↓	[14]
	Myocardial IRI	PI3K/AKT pathway ↑	[16]
	Renal IRI	PI3K/AKT pathway ↑	[18]
Promote apoptosis	Hepatic IRI	caspase-3/8-OHdG ↑	[21]
Promote angiogenesis	Cerebral IRI	YAP/TAZ pathway ↑	[15]
	Myocardial IRI	ERK/HIF-1α/VEGF pathway ↑	[17]
	Renal IRI	VEGFA pathway ↑	[20]
Inhibit fibrosis	Myocardial IRI	PI3K/AKT pathway ↑	[16]
	Renal IRI	α-SMA ↓	[19]
	Hepatic IRI	α-SMA ↓	[21]

Note: NRF2, nuclear factor erythroid 2-related factor 2; ARE, antioxidant response element; caspase-3, cysteine-dependent aspartate-specific protease-3; PI3K, phosphatidylin-ositol 3-kinase; ERK, extracellular signal-regulated kinase; HIF-α, hypoxia-inducible factor 1 α; VEGF, vascular endothelial growth factor; ELA, ELABELA; VEGFA, vascular endothelial growth factor A; MCP-1, Monocyte Chemoattractant Protein-1; ICAM-1, Intercellular Adhesion Molecule-1; MPO, Myeloperoxidase; α-SMA, α-Smooth Muscle Actin; 8-OHdG, 8-hydroxydeoxyguanosine.

## Data Availability

No new data were created or analyzed in this study. Data sharing is not applicable to this article.

## Abbreviations

The following abbreviations are used in this manuscript:

ELA	Elabela
IRI	Ischemia–reperfusion injury
PCI	Percutaneous coronary intervention
STEMI	ST-segment elevation myocardial infarction
ROS	Reactive oxygen species
mPTP	mitochondrial permeability transition pores
DAMPs	Damage-associated molecular patterns
TNF	Tumor necrosis factor
IL	Interleukin
TRAF1	TNF Receptor-Associated Factor 1
NF-κB	Nuclear Factor Kappa-light-chain-enhancer of Activated B cells
DOCA	Deoxycorticosterone Acetate
KLF15	Krüppel-like Factor 15
GSH	glutathione
GPX4	Glutathione peroxidase 4
FGF21	Fibroblast growth factor 21
ACE2	Angiotensin-converting enzyme 2
NRF2	Nuclear factor erythroid 2-related factor 2
ARE	Antioxidant response element
caspase-3	cysteine-dependent aspartate-specific protease-3
PI3K	Phosphatidylin-ositol 3-kinase
ERK	Extracellular signal-regulated kinase
VEGF	Vascular endothelial growth factor
α-SMA	α-Smooth Muscle Actin
MCP-1	Monocyte Chemoattractant Protein-1
ICAM-1	Intercellular Adhesion Molecule-1
Bcl-2	B-cell lymphoma 2
CTDSP1	C-terminal domain small phosphatase 1
FTH1	Ferritin heavy chain
CMVECs	Cardiac microvascular endothelial cells
HIF-1a	Hypoxia-inducible factor-1a
MDA	Malondialdehyde
4-HNE	4-hydroxynonenal
AKI	Acute kidney injury
CKD	Chronic kidney disease
Angpt1	Angiotpoietin 1
VEGFA	Vascular endothelial growth factor A
MPO	Myeloperoxidase
8-OHdG	8-hydroxydeoxyguanosine
